# Terahertz Metasurfaces Exploiting the Phase Transition of Vanadium Dioxide

**DOI:** 10.3390/ma16227106

**Published:** 2023-11-09

**Authors:** Meng Liu, Ruxue Wei, Jasmine Taplin, Weili Zhang

**Affiliations:** 1 College of Electronic and Information Engineering, Shandong University of Science and Technology, Qingdao 266590, China; liumeng@sdust.edu.cn; 2 School of Electrical and Computer Engineering, Oklahoma State University, Stillwater, OK 74078, USA

**Keywords:** terahertz, metasurfaces, vanadium dioxide, phase transition, dynamic modulation, external stimuli

## Abstract

Artificially designed modulators that enable a wealth of freedom in manipulating the terahertz (THz) waves at will are an essential component in THz sources and their widespread applications. Dynamically controlled metasurfaces, being multifunctional, ultrafast, integrable, broadband, high contrasting, and scalable on the operating wavelength, are critical in developing state-of-the-art THz modulators. Recently, external stimuli-triggered THz metasurfaces integrated with functional media have been extensively explored. The vanadium dioxide (VO_2_)-based hybrid metasurfaces, as a unique path toward active meta-devices, feature an insulator–metal phase transition under the excitation of heat, electricity, and light, etc. During the phase transition, the optical and electrical properties of the VO_2_ film undergo a massive modification with either a boosted or dropped conductivity by more than four orders of magnitude. Being benefited from the phase transition effect, the electromagnetic response of the VO_2_-based metasufaces can be actively controlled by applying external excitation. In this review, we present recent advances in dynamically controlled THz metasurfaces exploiting the VO_2_ phase transition categorized according to the external stimuli. THz time-domain spectroscopy is introduced as an indispensable platform in the studies of functional VO_2_ films. In each type of external excitation, four design strategies are employed to realize external stimuli-triggered VO_2_-based THz metasurfaces, including switching the transreflective operation mode, controlling the dielectric environment of metallic microstructures, tailoring the equivalent resonant microstructures, and modifying the electromagnetic properties of the VO_2_ unit cells. The microstructures’ design and electromagnetic responses of the resulting active metasurfaces have been systematically demonstrated, with a particular focus on the critical role of the VO_2_ films in the dynamic modulation processes.

## 1. Introduction

Metasurfaces have revealed unprecedented flexibility to arbitrarily manipulate the electromagnetic waves at a subwavelength scale, emerging into an essential platform for terahertz (THz) control [1]. The deep subwavelength characteristics endow metasurfaces to realize the ultra-compact configuration and mitigate the fabrication complexity of the three-dimensional counterparts. Metasurfaces not only boost the performance of traditional devices but can also engineer optical responses which cannot occur from traditional devices, including but not limited to the negative index [2,3], super lensing [4,5], invisibility cloaking [6,7], and perfect resonance absorption [8,9,10]. This is especially true at THz frequencies where functional devices are scarce due to a large mismatch between the THz wavelengths and the characteristic dimensions of molecules and crystal lattices.

**Figure 1:**
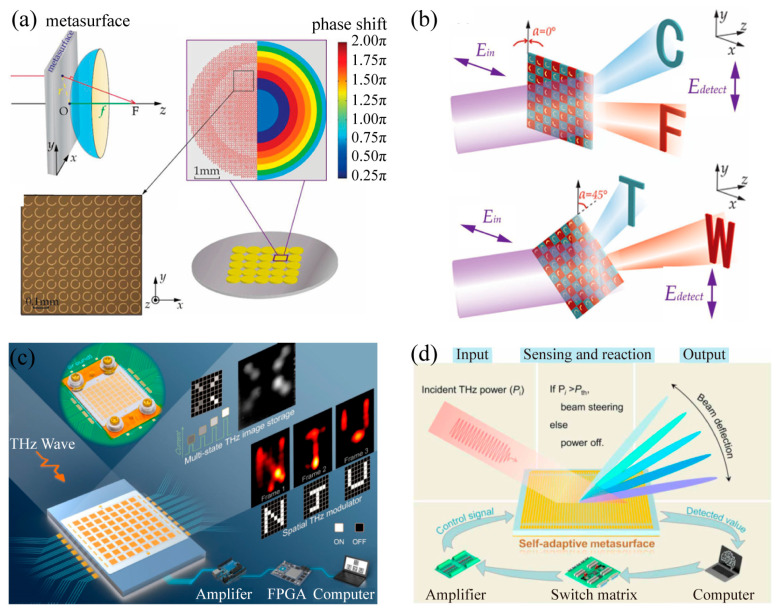
Representative applications of THz metasurfaces. (**a**) Design of a metasurface-based flat-lens array [5]. “Reprinted/adapted with permission from Ref. [5]. 2015, WILEY-VCH Verlag GmbH & Co. KGaA, Weinheim”. (**b**) Schematic illustration of the polarization and frequency selective meta-hologram’s functionality [11]. “Reprinted/adapted with permission from Ref. [11]. 2017, WILEY-VCH Verlag GmbH & Co. KGaA, Weinheim”. (**c**) Schematic illustration and optical image of the proposed VO_2_-based THz spatial light modulators [12]. “Reprinted/adapted with permission from Ref. [12]. 2022, WILEY-VCH Verlag GmbH & Co. KGaA, Weinheim”. (**d**) Illustration of the VO_2_-based THz self-adaptive metasurface for self-adaptive beam steering [13]. “Reprinted/adapted with permission from Ref. [13]. 2022, American Association for the Advancement of Science”.

At present, THz metasurfaces have been widely applied to develop functional meta-devices, including lenses (Figure 1a) [5], holographic plates (Figure 1b) [11], spatial light modulators (Figure 1c) [12], optical smart devices (Figure 1d) [13], waveplates [14,15,16], rotators [17,18], invisibility cloaking [19], and achromatic devices [20,21]. The electromagnetic responses of conventional THz metasurfaces made of metallic or dielectric microstructures are determined by the lattice configuration and material composition, which are unchangeable once the samples are fabricated. Passive and single-functioning THz metasurfaces exhibit numerous limitations in practical applications, such as high-speed communications, imaging, display, and optical computing. To mitigate this dilemma, dynamically controlled THz metasurfaces have been proposed and developed to incorporate active control of THz radiation. Summing up the previous work, there have been two approaches to dynamically tailor the microscopic resonance of microstructures and the macroscopic electromagnetic responses of THz metasurfaces. One approach is to deform the shape of the resonators by flipping [22], rotating [23], shrinking or enlarging [24,25], or bending [26,27,28,29,30,31,32,33] the microstructures. The microstructure reconfiguration enables dynamic control of its resonance and a real-time modulation of the electromagnetic responses. However, sample fabrication turns out to be more complex when this method was expanded to high frequencies. In addition, the low modulation rate also greatly limited its practical applications. The other approach is to tailor the dielectric properties of the substrates [34,35,36,37,38,39,40]. In this method, in addition to the dielectric properties of the large thickness substrate material, which are difficult to modify, its high dielectric constant is also unfavorable to the tuning of metasurfaces. Moreover, modifying the dielectric properties of the thick substrate can significantly reduce the transmission of the device. Therefore, two-dimensional (2D) functional materials, such as graphene [41,42,43,44], black phosphorus [45,46], silicon on sapphire [47], Ge-Sb-Te alloy [48,49], Dirac semi-metal [50], semiconductor [51], superconductor [52], and vanadium dioxide (VO_2_) [53,54,55,56,57], are promising in developing actively controlled meta-devices. These continuous or patterned 2D materials are usually integrated with metallic metasurfaces either being sandwiched by a spacer or directly patterned as metasurfaces to actively manipulate the THz waves under the excitation of heat, electricity, light, or intensive THz field.

The VO_2_ film is an attractive candidate showing dynamically controlled dielectric properties under external stimuli. In 1959, Mott predicted that VO_2_ experienced a reversible phase transition between the insulator and metal using the band theory [58]. Then, VO_2_ turned to be the most interesting compound in the V-O system as its transition temperature *Tc* (approximately 65~68 °C) nears room temperature. The phase transition of the VO_2_ film can be realized via thermal, electrical, and optical excitations that are accompanied not only by a drastic conductivity change exceeding four orders of magnitude, but also by the remarkable modification of electrical and optical properties at all wavelengths [54,59,60]. Furthermore, the phase transition of the VO_2_ film is hysteretic and the phase transition temperature between the heating and cooling branch is different, which provides a platform for the development of optical memory and storage devices. Most importantly, the phase transition temperature *Tc* and the hysteresis loop temperature width can be adjusted via doping [61,62,63,64,65,66,67], ion irradiation [68], stressing [69], and nano-structuring [70,71], realizing a phase transition temperature closer to room temperature. Compared with the most widely employed phase change materials including GeTe, Ge_2_Sb_2_Te_5_, GeSb_2_Te_4_, and AgInSbTe, VO_2_ exhibits prominent advantages in practical applications as it can operate at room temperature with a lower switching threshold [72]. The phase transition temperature of the GST materials can reach up to 300 °C, which is much higher than that of VO_2_. Clearly, the transition of VO_2_ is more convenient, efficient, energy-saving, and low cost. Thus, the VO_2_ films have been extensively pursued and selected as a suitable candidate in developing THz dynamic metasurfaces to achieve the active modulation of THz radiation in terms of amplitude, phase, and polarization. The proposed VO_2_-based THz metasurfaces are usually composed of metal metasurfaces and VO_2_ film. The near-field interactions between the individual resonators and the electro-magnetic responses of these hybrid metasurfaces can be actively tuned via the highly contrasting conductivity jump under the external stimuli. In particular, the continuous or structured VO_2_ films should be arranged in a location where the resonant electromagnetic energy of the metal microstructure is concentrated. In addition, certain THz metasurfaces are purely composed of the VO_2_ film microstructures, taking advantages of the resonant and phase transition properties of the VO_2_ microstructures to actively manipulate the terahertz waves.

In this review, we present recent advances in dynamically controlled THz metasurfaces exploiting the VO_2_ phase transition. First of all, we will introduce the phase transition of the VO_2_ film categorized according to the external stimuli. Then, characterization of the VO_2_ films by use of THz time-domain (THz-TDS) spectroscopy will be described. Next, we will present the recent progress on the VO_2_-based THz metasurfaces, as classified and organized in the categories of stimuli. For each type of excitation, four design strategies are employed to realize the external stimuli-triggered VO_2_-based THz metasurfaces, including switching the transreflective operation mode, controlling the dielectric environment of metallic microstructures, tailoring the equivalent resonant microstructures, and modifying the electromagnetic properties of the VO_2_ unit cells. The microstructures design and electromagnetic responses of the resulting active metasurfaces will be systematically demonstrated, where we will particularly focus on the critical role of the VO_2_ films in the dynamic modulation processes. Finally, we will present a conclusion and prospect on the VO_2_-based THz metasurfaces.

## 2. The Phase Transition of the VO_2_ Films

The insulator-to-metal transition behaviors accompanied by a rapid reversible phase transition in the V-O systems have gained substantial attention. The V-O systems include but are not limited to VO, V_2_O_3_, V_3_O_5_, V_4_O_7_, V_5_O_9_, V_6_O_11_, V_7_O_13_, V_8_O_15_, VO_2_, and V_2_O_5_ [73]. Compared with other V-O systems, the transition temperature *Tc* of VO_2_ is not only most close to room temperature, but can also be modulated from 70 to 20 °C via chemical element doping [63] or lattice epitaxial stress [64]. Therefore, VO_2_ exhibits significant potentials in practical applications, including active polarizers [68], absorbers [41,74], lens [75,76], holography [56], mirrors [54], asymmetric transmission devices [55], memory and storage devices [77,78,79], intelligent windows [63], and radiation sources [80].

**Figure 2:**
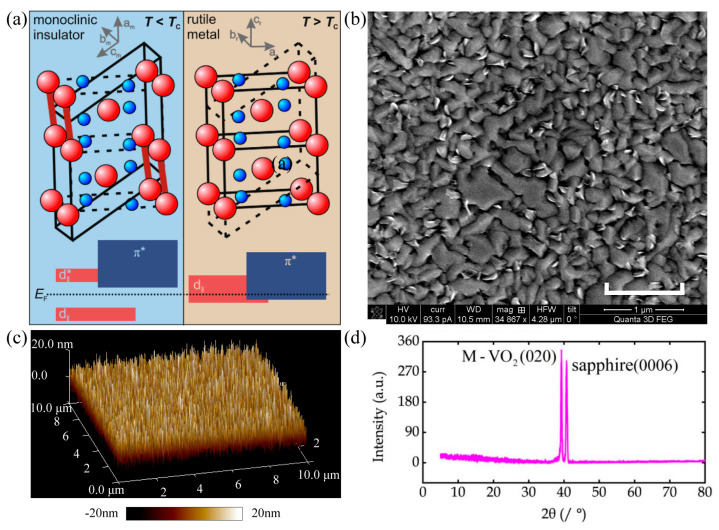
(**a**) Comparison of monoclinic insulating (**left**) and rutile metallic VO_2_ (**right**): top: crystallographic structures, the unit cells are indicated by the solid lines. The vanadium atoms are represented by the red balls and the oxygen atoms are marked blue. (**Bottom**): schematic representation of the VO_2_ electronic band structure in the vicinity of the Fermi level [81]. “Reprinted/adapted with permission from Ref. [81]. 2015, Elsevier Ltd.”. (**b**) Scanning electron microscopic image for a 150 nm VO_2_ film grown on 500 μm c-cut sapphire substrate. The scale bar corresponds to 1 μm [54]. “Reprinted/adapted with permission from Ref. [54]. 2020, WILEY-VCH Verlag GmbH & Co. KGaA, Weinheim”. (**c**) Atomic force microscopic measurement [54] and (**d**) X-Ray diffraction measurement for the sample shown in panel (**b**) [54].

VO_2_ exhibits many different structures under various growth conditions, in which the M1 and R phase are most common due to their reversible transition near the room temperature, as shown in Figure 2a [81]. In the monoclinic band structure, the *d*_||_ band is divided into two energy bands *d*_||_ and *d*_||_* with a forbidden gap that occurs between the *d*_||_ and π* band. The Fermi level falls within the forbidden band with approximately a 0.7 eV forbidden bandwidth, which makes VO_2_ insulative. On the other hand, in the rutile phase band structure, the Fermi level falls on the π* band and the *d*_||_ band simultaneously, thus showing an electric conductivity as high as that of a metal [82]. In the VO_2_ system, the coupling of lattice, charge, spin, and orbital makes the VO_2_ reveal abnormal physical phenomena in optical, electric, thermal, and other properties near the phase-transition point. Presently, the phase-transition mechanism of VO_2_ is still controversial and an in-depth understanding of the phase-transition mechanisms and its modulation is still in urgent need.

Since the bulk of VO_2_ is fragile under the multiple phase transition cycles, the durable VO_2_ thin film exhibits promising applications for electrical and optical components. The fabricated VO_2_ films are usually characterized via scanning electron microscopy, atomic force microscopy, and X-ray diffraction. A representative study has been conducted by M. Liu and co-workers [54], where the fabricated 150 nm thick crystalline VO_2_ films consisted of a random, semi-continuous network of voids and nanoparticles with a mean diameter of approximately 500 nm, as shown in Figure 2b. According to the atomic force microscopy shown in Figure 2c, the root-mean-square surface roughness of the films is *R_q_* = 5.54 nm, which is small enough compared to their thickness. Furthermore, the room-temperature X-ray diffraction measurements reveal intensity peaks at angles 2*θ* of 39.2° and 41.3°, as shown in Figure 2d, indicating the crystalline VO_2_ and sapphire, respectively. The fabricated VO_2_ films exhibited a single monoclinic structure with a preferred orientation of (020) at room temperature. The above characterization results indicated that the continuous VO_2_ film is uniform and highly oriented with fewer defects.

### 2.1. Stimuli Category for Phase Transition of the VO_2_ Films

The strongly correlated electric material VO_2_ experiences an insulator–metal phase transition, which can be triggered by external physical excitations, including thermal, electricity, light, etc. In the following, we introduce recent progress in the phase transition of the VO_2_ films, categorized according to the stimuli.

#### 2.1.1. Thermally Triggered Phase Transition of the VO_2_ Film

**Figure 3:**
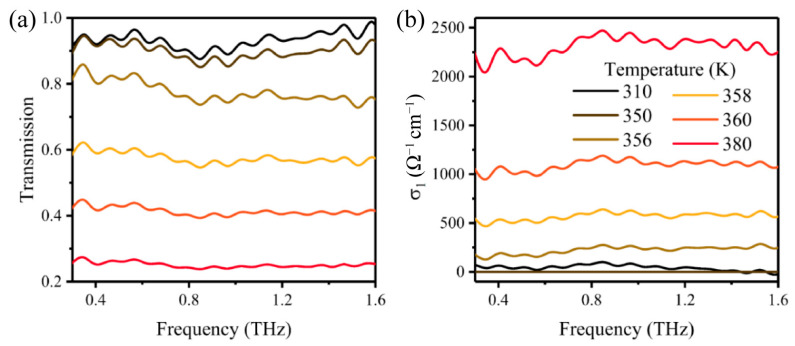
THz responses of the VO_2_ thin film with operation temperature as a parameter. (**a**) Transmission spectra of the VO_2_ film at various temperatures. (**b**) Temperature dependence on the conductivity of the VO_2_ film at THz frequencies [59]. “Reprinted/adapted with permission from Ref. [59]. 2018, WILEY-VCH Verlag GmbH & Co. KGaA, Weinheim”.

Thermal excitation is the most common and effective method to activate the phase transition of VO_2_ by globally heating the whole sample [54,59,60,61,66,70,71,83,84,85,86,87]. Along with phase transition, the electrical, optical, thermal, and magnetic properties of the VO_2_ films will be modified. In a previous experiment, Hood and co-workers demonstrated that the permittivity and conductivity of VO_2_ revealed a strong temperature dependence at millimeter frequencies when the excitation temperature passed through the phase transition temperature [88]. Then, Jepsen and co-workers measured the high-frequency conductivity during phase transition in a polycrystalline VO_2_ film deposited on a glass substrate by use of THz-TDS for the first time. This work demonstrated that the conductivity of the polycrystalline VO_2_ film was strongly modulated during phase transition in a broad THz frequency range [84]. Furthermore, this work indicated that THz-TDS has developed to be a powerful platform for the characterization and extraction of material parameters at high frequencies without electrical contacts to the sample. Since then, an abundance of follow-up THz spectroscopy studies on the VO_2_ films have been reported [54,59,60,61,66,70,71,85,87]. Consequently, studies on single crystalline VO_2_ films were also carried out. Mandal and co-workers fabricated an epitaxial VO_2_ film on a c-cut sapphire substrate, which was highly oriented and exhibited single crystalline properties [86]. They consequently carried out a spectroscopy study on the single crystalline VO_2_ film across the phase transition with an amplitude modulation depth reaching 85%. The results indicated that the single crystalline VO_2_ film with a highly oriented performance exhibited greater conductivity modulation capacity compared to the polycrystalline counterparts. Recently, Lu and co-workers performed a systematic study on the phase transition of a 90 nm thick VO_2_ film deposited on a c-cut sapphire. Figure 3a,b illustrates the THz transmission and conductivity spectra of the VO_2_ film with operation temperature as a parameter [59]. The measured temperature-dependent transmission and conductivity of the VO_2_ film revealed the insulator-to-metal phase transition in the temperature range of 310 to 380 K. The transmission in the frequency range of 0.3 to 1.6 THz at room temperature 310 K was more than 90%, corresponding to a low conductivity and an insulating monoclinic crystal structure of VO_2_. The transmission decreased dramatically as the film was heated across the phase transition temperature, corresponding to a similar dramatic increase in conductivity. At temperatures of 350–360 K, the VO_2_ film had a mixture of the insulating monoclinic crystal structure and the metallic rutile crystal structure. By further increasing the operation temperature to 380 K, the transmission dropped to ~0.25 and its conductivity approached 2.25 × 10^3^ S/cm in the whole frequency range, indicating that the VO_2_ film was fully switched to the metallic rutile crystal structure (right inset).

However, there is still a remaining transmission of more than 0.25 even though the VO_2_ film was in its metallic state, as shown in Figure 2a. This occurs from the skin depth of the THz wave being much greater than the thickness of the VO_2_ film; thus, the metallic VO_2_ film has a limited level of transmission to the THz wave [87].

Furthermore, with the increase in working temperature, the VO_2_ film will transition from a dielectric state at room temperature to a metallic state at high temperatures and the phase transition would occur more dramatic around the phase transition temperature. Based on the interference cancellation and impedance matching effects, the deep subwavelength-thick VO_2_ films have achieved perfect absorption [74] and antireflection [61,69].

#### 2.1.2. Electrically Triggered Phase Transition of the VO_2_ Films

**Figure 4:**
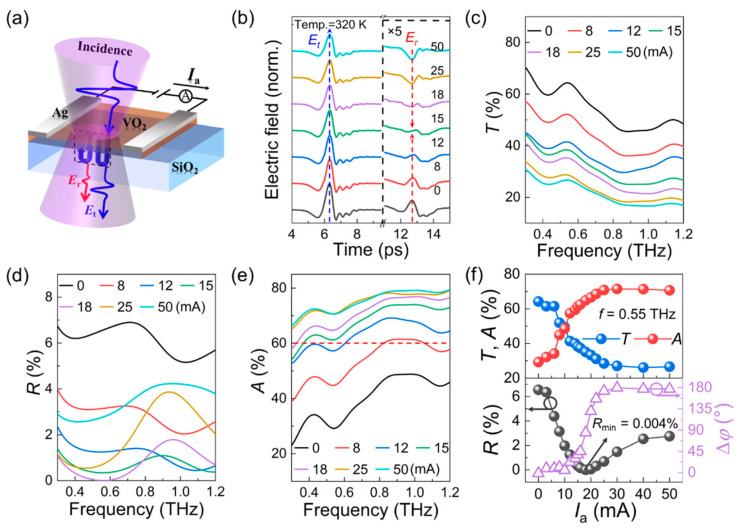
(**a**) Schematic diagram of actively electro-controlled VO_2_ film device for THz transmission (*T*) and reflection (*R*) modulation. (**b**) Experimental THz time-domain signals of a 75 nm thick VO_2_ thin film under different applied electric current excitations (0, 8, 12, 15, 18, 25, and 50 mA, respectively) at 320 K; the reflection echo pulses (*E*_r_) are normalized by peak amplitudes of the main pulses (*E*_t_) and multiplied by five times; a blue dashed line with an arrow indicates that *E*_t_ decreases monotonically; and two red dashed lines with arrows indicate that *E*_r_ decreases to a minimum value and then increases, but with the reversal of pulse shape with applied current. Corresponding THz (**c**) *T*, (**d**) *R*, and (**e**) absorption (A) intensity spectra; a red dashed line indicates *A* = 60%. Experimental *T*, *R*, *A*, and reflection (**f**) phase shift, seen in purple, (Δ*φ*) as a function of applied electric current at 0.55 THz. R reaches the minimum value *R*_min_∼0.004% at 0.55 THz under the current of 18 mA [93]. “Reprinted/adapted with permission from Ref. [93]. 2022, American Chemical Society”.

In contrast to the global heating of thermal excitation, the electrically triggered VO_2_ film activates the phase transition by inducing local heating precisely as the current flows through the VO_2_ film or metal wires, which shows certain advantages in energy saving and device integration. From the practical application point of view, electric excitation is a convenient and low-cost approach to activate the phase transition of the VO_2_ films. There have been extensive studies on the electrically triggered phase transition of the VO_2_ films, including electric field gating-induced phase transition [89,90,91,92] and electrical current heating-induced phase transition [93,94,95,96]. In electric field gating, the phase transition of the VO_2_ film was resulted from the carrier injection from the gate into the VO_2_ channel. While in electrical current heating, the phase transition of the VO_2_ film was resulted from the Joule thermal effect of the electric current. In 2022, Ren and co-workers demonstrated an electrical current-controlled THz modulator by use of the VO_2_ thin film [93]. As shown in Figure 4a, the VO_2_ and quartz layers had a thickness of 75 nm and 500 μm, respectively. The strip-shaped silver electrodes with a separation gap of 3 mm were deposited on the VO_2_ film. The THz beam excited the modulator at normal incidence in the THz TDS system. Stimulating the VO_2_ film via a direct current (~mA) would create a voltage bias between the electrodes, producing abundant amounts of micrometer conductive filaments. This led to phase transition and a remarkable conductivity increase in the VO_2_ film. Subsequently, the ohmic thermal effect of the current would further promote the phase transition and increase the conductivity gradually, as shown in Figure 4b. On the contrary, when the excitation current was cancelled, the conductive filaments and ohmic thermal effect disappeared immediately. As a result, the metallic VO_2_ returned back to its insulating medium state. Based on the current-induced phase transition in the VO_2_ film, the broadband (0.3–1.2 THz) transmission shown in Figure 4c, the reflection shown in Figure 4d, the absorption shown in Figure 4e, and the phase delay shown in Figure 4f, one can confirm that the amplitudes of the THz signals were dynamically modulated. In addition, the amplitude and phase responses of the VO_2_ film at 0.55 THz are provided in Figure 4f. In the top panel, the transmission and absorption decrease and increase respectively with the increase of applied current, and the phenomenon reaches saturation when the current is equal to 30 mA. As shown in the bottom panel, the reflection coefficient also changes similarly. As shown in the bottom panel, with the increase of the excitation current, the reflection decreases first and then increases, reaching a minimum value of ~0.004% when the applied current is 18 mA. Most importantly, the phase shift of the reflected wave changes from 0° to 180° around this reflection minimum, indicating that the VO_2_ film changes from the dielectric state to metal state. Thus, the reflection component could not only achieve a high modulation depth of up to 99.9% with a π phase shift, but could also initiate a stable multibit modulation. Additionally, the antireflection condition could be actively and intelligently initiated by using a feedback loop of “THz-electro-THz” system. This was the first demonstration of a current-controlled THz reflection modulator based on the VO_2_ film.

#### 2.1.3. Optically Triggered Phase Transition in the VO_2_ Films

**Figure 5:**
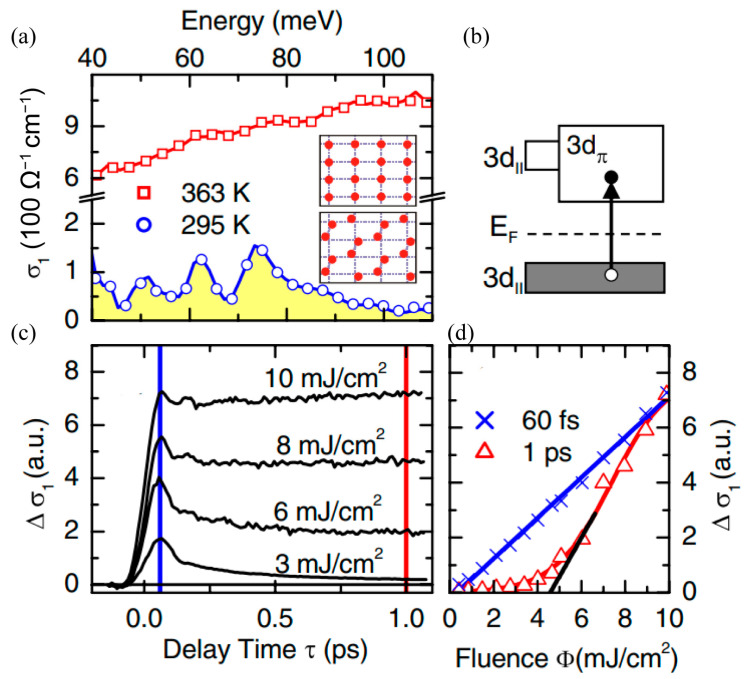
(**a**) Mid-IR conductivity spectra of VO_2_ at *T*_L_ = 295 K (blue circles) and 363 K (red squares). Inset: V sublattice of the *R* (upper part) and *M*_1_ (lower part) phase. (**b**) Schematic of photodoping in the insulating band structure of VO_2_. (**c**) Spectrally integrated transient change in the THz conductivity after excitation at various fluences (*T*_L_ = 295 K). (**d**) Fluence dependence on Δσ_1_(*τ*) at *τ* = 60 fs (blue crosses) and 1 ps (red triangles). A linear fit to the latter graph extrapolates to a critical fluence *Φ*_c_ = 4.6 mJ/cm^2^ [97]. “Reprinted/adapted with permission from Ref. [97]. 2007, The American Physical Society”.

Optical excitation with a photon energy larger than the insulation phase gap of approximately 0.7 eV has been verified to be another effective approach to promote the phase transition of the VO_2_ films [97,98,99]. The photo-excited VO_2_ phase transition is a synthesis result of a light-excited carrier phase transition and a photo-thermal phase transition. In the case of continuous laser pumping, local photo-thermal effects can stimulate VO_2_ to achieve a millisecond phase transition [100]. However, the phase transition of the VO_2_ film under femtosecond pulse excitation is mainly resulted from the light-excited carrier phase transition, and the phase transition caused via photo-thermal excitation is very weak. In 2007, Kubler and co-workers demonstrated an insulator-to-metal phase transition in the VO_2_ film by using femtosecond laser pulses [97]. The polycrystalline VO_2_ film was grown on a diamond substrate with a thickness of 120 nm. It was characterized by using an optical-pump THz-probe system, which enabled them to optically modify and numerically extract the complex conductivity in broadband frequency range. The real part of conductivity of the VO_2_ sample is shown in Figure 5a. When the operation temperature was increased from 295 K to 363 K, which was beyond the critical temperature, the phase transition occurred in the VO_2_ film accompanied with a conductivity increase of 1~2 orders of magnitude in the concerned frequency range. On the other hand, ultrafast transition of electrons from the valence to the conduction band can be achieved via femtosecond laser excitation, as shown in Figure 5b. Figure 5c illustrates the time delay dependence on conductivity with various pump fluence. For the low excitation intensity of 3 mJ/cm^2^, light-excitation conductivity exhibited a rapid increase accompanied by a sub-picosecond decay. With the increase in excitation fluence, the photo-excited conductivity increased gradually. It also featured an increasing long-lived background conductivity that remained constant within a time window of 10 ps. As a result, the insulating VO_2_ turned to a metallic state gradually throughout the photo-excitation process. With a further increase in the excitation fluence to 10 mJ/cm^2^, the photo-excited conductivity reached a large value quickly with little attenuation and remained constant for a long time. Conductivity increasement resulted from the excitation density of 10 mJ/cm^2^, improved by orders of magnitude compared to the increase that resulted from the excitation density of 3 mJ/cm^2^. At this time, the initial insulating VO_2_ film has been converted to the metallic phase completely. The above results reveal that the time for VO_2_ switching from dielectric state to metallic state does not change significantly for various pump intensities, as marked by the blue line. This shows that the phase transition rate of photoexcited VO_2_ is basically not affected by the pump intensity. Additionally, the pump fluence dependence on conductivity for two specific early delay times of 60 fs and 1 ps are shown in Figure 5d. The quasi-instantaneous signal for the delay time of 60 fs scaled linearly with the pump fluence since the conductivity in this case was resulted from direct light-injected carriers. On the contrary, the quasi-instantaneous signal for the delay time of 1 ps presented a threshold performance: the THz conductivity disappeared below 4.6 mJ/cm^2^ and then it increased almost linearly with the increasing pump fluence, exhibiting a cooperative ultrafast insulator-metal transition.

### 2.2. THz Spectroscopy Characterization Modalities of the VO_2_ Films

**Figure 6:**
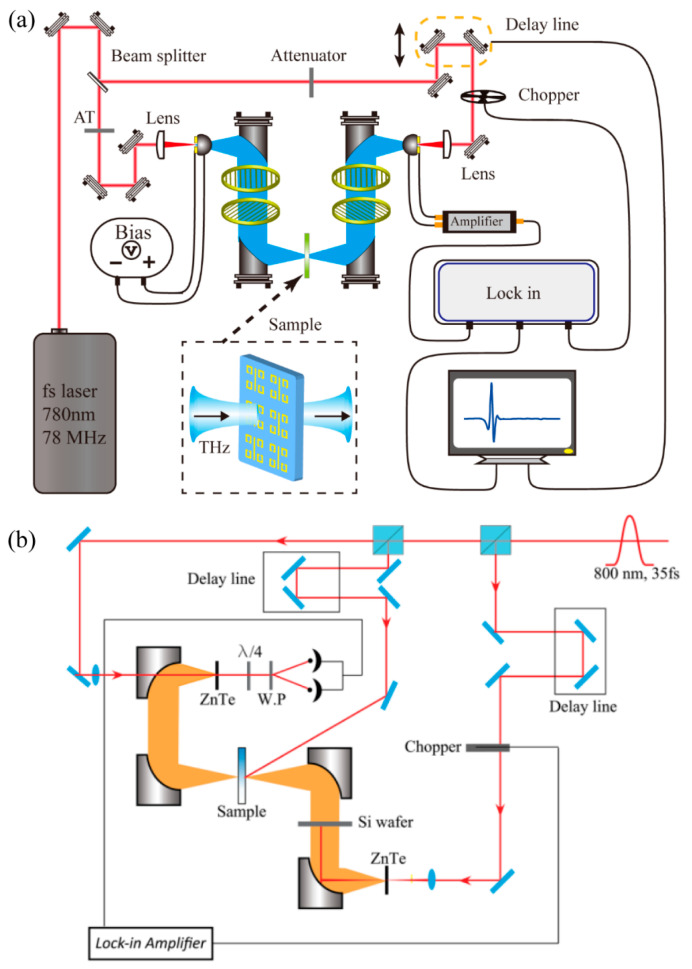
Schematic diagram of a representative THz-TDS system used to characterize the spectral response of the VO_2_ films with external excitation. (**a**) Photoconductive antenna-based THz-TDS system [102]. “Reprinted/adapted with permission from Ref. [102]. 2018, IOP Publishing Ltd.”. (**b**) Optical-pump THz-probe spectroscopy system [59]. “Reprinted/adapted with permission from Ref. [59]. 2018, WILEY-VCH Verlag GmbH & Co. KGaA, Weinheim”.

As shown in Figure 6a, conventional THz-TDS systems were employed to record the spectra responses of the VO_2_ films [54,59,60,70,71,85,86,87,93]. The femtosecond laser output was divided into two beams with identical intensity and then they were guided and focused onto the photoconductive transmitter antenna and photoconductive receiver antenna independently. One beam was applied to generate the THz pulse by stimulating the biased transmitter antenna. The other beam behaved as a gate for the photoconductive receiver antenna. The temporal shape of the THz transient was collected by gradually controlling the relative delay between the THz pulse and the gate laser pulse while recording the voltage or current data in the external circuit which were induced by the THz field. The prepared VO_2_/substrate was firmed on a hot plate which had an aperture at the center for the transmission measurement. All the measurements were taken in a dry environment with a THz beam illuminating the sample at normal incidence. The above photoconductive antenna-based THz-TDS system is suitable for the characterization of the VO_2_ film triggered by heat, electricity, and continuous light. However, for the VO_2_ film triggered by pulsed lasers, the optical-pump THz-probe spectroscopy system, as shown in Figure 6b, would be implemented [59,85,97,101]. The Ti: sapphire regenerative amplifier with a 35 fs pulse width, a central wavelength of 800 nm, and a repetition rate of 1 kHz was utilized as the pumping source of the THz system. The laser output was divided into three beams. The first beam was applied for the emission of the THz wave, the second beam was used for the detection of the THz waves, and the third laser beam was the most powerful one applied to excite the sample. In optical-pump THz-probe spectroscopy, the THz radiation was generated and detected by the optical rectification effect and the electro-optical detection effect in zinc telluride (ZnTe) crystals, respectively. The optical-pump THz-probe spectroscopy system was demonstrated to be an advantageous modality in VO_2_ characterization, as it not only provided an intensive pump energy, but also revealed the dynamic process resulting from the external light excitation.

## 3. VO_2_-Based THz Metasurfaces

**Figure 7:**
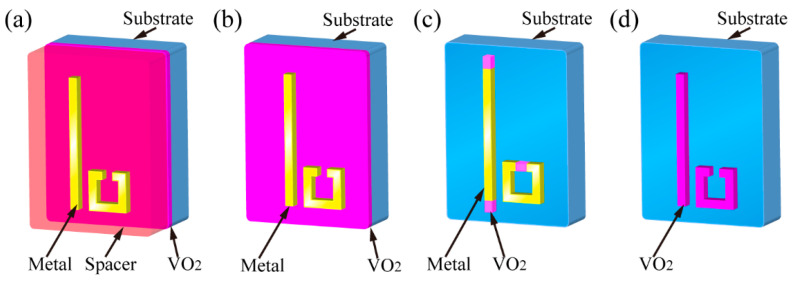
Schematics of four types of design strategies to implement external stimuli-triggered THz metasurfaces based on the VO_2_ films. (**a**) Switching the transreflective operation mode realized by integrating a continuous VO_2_ film with metallic metasurfaces and separated by spacers. (**b**) Controlling the dielectric environment of the meta-structure realized by inserting a continuous or patterned VO_2_ film between the substrate and meta-structure layer. (**c**) Tailoring the equivalent resonant microstructure achieved by patterning the structured VO_2_ film to lengthening, shortening, and connecting the meta-structure. (**d**) Modifying the electromagnetic property of the VO_2_ microstructures, which is composed of the VO_2_ microstructure array only.

Metasurfaces have opened up a unique route toward THz wave control. A considerable number of functional devices have been generated via this powerful platform. However, metasurface-based devices are often limited to operating with a single function, thus limiting their applications where multi-functional devices are needed. To mitigate this difficulty, the introduction of active materials into metasurfaces is undoubtedly an optimal solution. Accordingly, ultra-thin VO_2_ films grown on transparent substrates to the THz radiation, such as sapphire, quartz, and silicon, are promising in developing active THz metamaterials. The drastic conductivity change in the VO_2_ film during phase transition exerts a strong influence on the THz transmission, reflection, and absorption. Thus, by introducing VO_2_ films into metasurfaces, the resonance of the subwavelength microstructures can be actively modified by use of the externally controlled phase transition of the VO_2_ film. This will lead to dynamic THz metasurfaces under the excitation of heat, electricity, and light. For each of these excitations, four design strategies were applied to implement the external stimuli for the VO_2_-based THz metasurfaces, including (1) switching the transreflective operation mode, as shown in Figure 7a. This can be implemented by integrating a continuous VO_2_ film with metallic metasurfaces sandwiched by spacers; (2) controlling the dielectric environment of metallic microstructures, as shown in Figure 7b, which can be performed by inserting a continuous or patterned VO_2_ film between the substrate and the meta-structure layer; (3) tailoring the equivalent resonant microstructure, as shown in Figure 7c, which can be achieved by patterning the structured VO_2_ film to modify the design of the meta-structures; and (4) modifying the electromagnetic property of the VO_2_ microstructures, as shown in Figure 7d. This metasurface is composed of the VO_2_ microstructure array only. In the following sections, we will review these four types of VO_2_-based metasurfaces according to their classification of excitation mode.

### 3.1. Thermally Triggered VO_2_ Film-Based THz Metasurfaces

**Figure 8:**
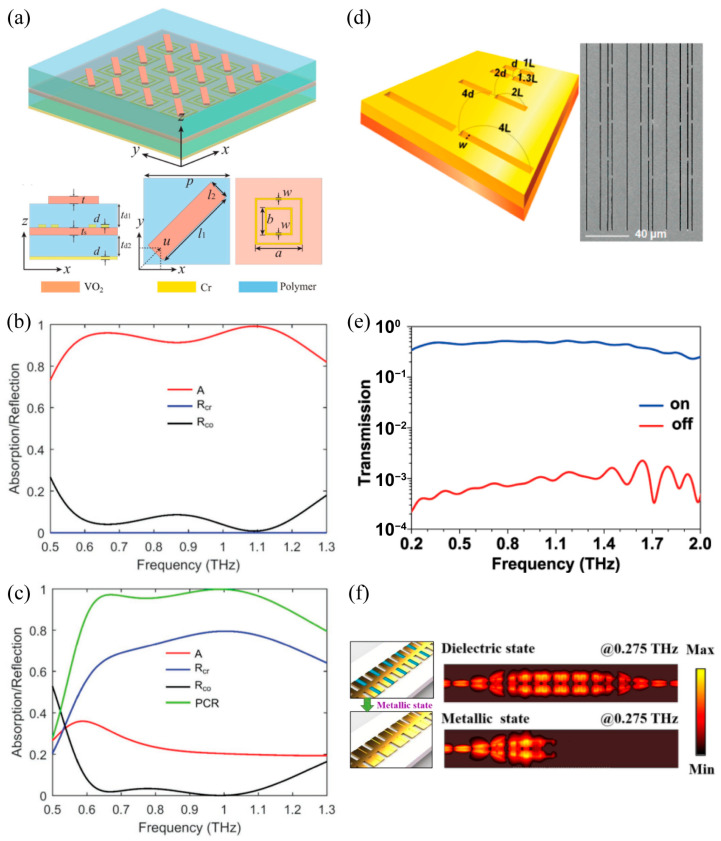
(**a**) Schematic of the VO_2_-integrated metasurface for diversified functionalities switch. The unit cell of the metasurface is composed of multiple layers. (**b**) Simulated absorption, co-polarized reflection, and cross-polarized reflection at normal incidence when VO_2_ was in the insulating state with *σ* = 200 S m^−1^. (**c**) Simulated absorption, co-polarized reflection, cross-polarized reflection, and PCR at normal incidence when VO_2_ was in its fully metallic state with *σ* = 200,000 S m^−1^ [103]. “Reprinted/adapted with permission from Ref. [103]. 2018, WILEY-VCH Verlag GmbH & Co. KGaA, Weinheim”. (**d**) Ultra-broadband active metamaterial at THz frequencies: schematics for broadband gold resonator patterns on a VO_2_ thin film (**left**) and scanning electron microscopy image of a nanoresonator pattern sample (350 nm width and 50, 65, 100, and 200 µm lengths with 3, 7, and 13 µm separations, respectively) (**right**). (**e**) Logarithmic plot for transmittances of the ultra-broadband resonator on the VO_2_ film at frequencies of 0.2–2.0 THz for 305 K (blue lines) and 375 K (red lines) [87]. “Reprinted/adapted with permission from Ref. [87]. 2010, American Chemical Society”. (**f**) Schematic of the tunable spoof surface plasmon transmission line at different states and the simulated near-electric field distribution on an observation plane that is 2 µm above the transmission line in the dielectric and metallic states [105]. “Reprinted/adapted with permission from Ref. [105]. 2019, IEEE Xplore”.

Temperature-induced phase transition of the VO_2_ film is accompanied not only by a drastic conductivity change exceeding four orders of magnitude, but also by the remarkable modification of electrical and optical properties. Therefore, the VO_2_-based THz metasurfaces can administer active control of the THz wave via thermal excitation. First, we summarize the thermally triggered THz metasurfaces that switch the operation mode by integrating a single continuous VO_2_ film [50,103]. Since the insulating and metallic VO_2_ films exhibit high transmission as well as reflection to the THz incidence, the combination of the VO_2_ film and THz metasurfaces can compose multifunctional meta-devices with switchable operating modes. As seen in Figure 8a, a continuous VO_2_ film was inserted between the multilayer composite metasurfaces [103]. At room temperature, the bottom chrome plate and chrome double square ring resonators constituted a polarization-insensitive absorber. The absorptivity observed was over 90% and in the frequency range of 0.562–1.232 THz, as shown in Figure 8b. Increasing the operation temperature well above the phase transition temperature, the inserted continuous VO_2_ film and VO_2_ bars formed a reflective half-wave plate. The operating bandwidth of this half-wave plate with a reflectance rate above 60% could exceed 0.49 THz, as shown in Figure 8c. This VO_2_-based hybrid metasurface can be converted from the absorber to the half-wave plate, producing a multi-functional THz meta-device. Based on a similar design strategy, a THz metasurface with a switchable transmission and reflection was also devised by using the phase transition of the VO_2_ film, which was accompanied with the switching of asymmetric transmission and polarization conversion simultaneously [104]. Furthermore, this approach could also implement the switching between conventional and coherent absorbers based on the phase transition of the continuous VO_2_ film [50]. In the above design strategy of operation mode switching, the VO_2_ film was far away from the microstructure and it exerted little influence on the resonance of the microstructure. During the phase transition, the continuous VO_2_ film only behaved as a thin film with high transmission or high reflection.

Subsequently, we will review the second strategy of the thermally triggered THz metasurface: controlling the dielectric environment of the metallic microstructure by combining the VO_2_ continuous film [87,106,107] or VO_2_ patterns [105,108]. In contrast to the first design approach, patterning the metallic metasurface on the VO_2_ film directly or inserting the VO_2_ patches next to the microstructure would significantly reduce the gap between the two layers. In this situation, the microstructure resonance could be strongly influenced by the phase transition of the VO_2_ film and the electromagnetic response of the metasurface could be thermally modulated. As shown in Figure 8d, a gold multi-slot-resonator array was deposited onto the continuous VO_2_ film constructing a hybrid THz metasurface on a sapphire substrate which is not indicated here [87]. The transmission spectrum, as shown in Figure 8e, for insulating (305 K) VO_2_ revealed an ultra-broadband transparent response in the frequency range from 0.2 to 2 THz. By increasing the operation temperature well above the phase temperature (375 K), the transmission was significantly suppressed in the whole frequency range and the extinction ratio exceeded 1/1000. In this case, the vast extinction ratio resulted from the modified dielectric environment, which led to the phase transition of the VO_2_ film which disabled the resonance THz funneling through the gold slot in the whole frequency range. Subsequently, a similar VO_2_-based metasurface was demonstrated at the maximum modulation depth of 97.2% at 0.9 THz under thermal management [106]. Compared with the above single band THz metasurface, the dual-band dynamic metasurface filter has also been reported based on a similar design strategy [107], where the filter performance resulted from two kinds of microstructure resonance. However, the two filter bands were always manipulated simultaneously via phase transition of the continuous VO_2_ film, which cannot be dynamically manipulated separately. To mitigate this limitation, the VO_2_ patches, instead of continuous VO_2_ film, were inserted into a specific area of the hybrid metasurface to enable the independent control on the dual-band working mode. The phase transition of the VO_2_ patches modified the dielectric environment within the resonances to allow dynamic modulation. One benefit of this design strategy is the creation of dual-band metasurface absorbers with a controllable selected single absorption band. Simultaneously, the phase transition modified the dielectric environment of the selected resonance [108]. In addition to the above free-space THz metasurface modulators, a tunable THz spoof surface plasmon metasurface was initialized by patterning the VO_2_ patches onto the metallic corrugated strips, as shown in Figure 8f [105]. The heating-induced phase transition of the VO_2_ patches will modify the dielectric environment of the corrugated strips accordingly, contributing to the dynamically tuned dispersion and transmission properties of the spoof surface plasmon polaritons. Simulating near-electric field distribution demonstrated that the spoof surface plasmon polaritons at 0.275 THz could be transmitted effectively on the transmission line with VO_2_ in its insulating state; however, it was truncated absolutely when the integrated VO_2_ changed to its metallic state. The temperature excitation resulted in a switching transmission ratio on the spoof surface plasmon wave up to 12 dB from 0.22 to 0.28 THz and the maximum switching ratio exceeding 36 dB around 0.255 THz. In the above design strategy of controlling the dielectric environment of the resonant microstructure, the VO_2_ continuous film or VO_2_ film patches located in the resonance region of the metallic metasurface behaved as an external disturbance. The thermally induced phase transition of the VO_2_ film was applied to suppress the resonance of the metal microstructure.

**Figure 9:**
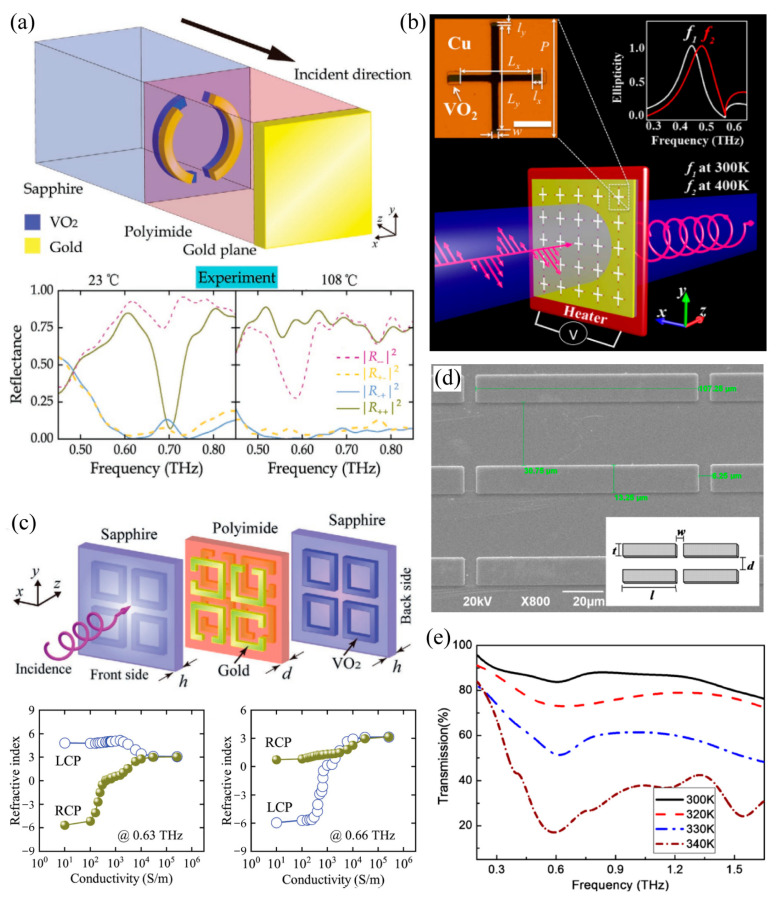
(**a**) Schematic of the switchable LCP/RCP mirror and the reflectance spectra measured at temperatures of 23 °C and 108 °C, respectively [54]. “Reprinted/adapted with permission from Ref. [54]. 2020, WILEY-VCH Verlag GmbH & Co. KGaA, Weinheim”. (**b**) Switchable THz quarter wave plate design and fabrication results. The top left inset is a microscopic image of one unit cell of the fabricated samples. The top right inset is the simulated ellipticities of the output THz waves, indicating that at both *f_1_* and *f_2_*, the output THz waves are circularly polarized [110]. “Reprinted/adapted with permission from Ref. [110]. 2015, Springer Nature”. (**c**) Top figures: metamaterial with switchable chirality. The unit cell is composed of pairs of identical and mutually twisted gold SRRs in parallel planes sandwiched in between the square VO_2_ rings. Bottom figures: refractive index for the RCP and LCP waves at 0.63 and 0.66 THz as a function of VO_2_ conductivity according to numerical simulations [53]. “Reprinted/adapted with permission from Ref. [53]. 2021, WILEY-VCH Verlag GmbH & Co. KGaA, Weinheim”. (**d**) SEM image of the VO_2_ cut-wire array. (**e**) Measured THz transmission curves for the cut-wire metamaterial at different device temperatures [60]. “Reprinted/adapted with permission from Ref. [60]. 2010, American Institute of Physics”.

Next, we will introduce the third strategy of thermally triggered THz metasurface with respect to tailoring the equivalent resonant microstructures by connecting metallic microstructures with the structured VO_2_ patterns. Hybrid metasurface microstructures that connect metallic resonators with the VO_2_ structure would exhibit a tunable resonance. This can be implemented by dynamically reconfiguring the equivalent resonant geometry via lengthening [54,75,109], shortening [110], and/or connecting [53,55,56] the metallic structure based on the phase transition of VO_2_. As shown in Figure 9a, an asymmetric split gold ring was patterned on an asymmetric VO_2_ ring on top of the sapphire substrate and they were separated from the gold mirror by a polyimide spacer to construct a THz chiral mirror [54]. The gap sizes and arc lengths differed with the shorter gold arc on the right and the shorter VO_2_ arc on the left when observed from the illumination side, leading to the opposite twist vector and chirality. The uncovered VO_2_ arc was applied to lengthen the shorter gold arc to a longer branch after the phase transition, producing a reserved chirality. The bottom panel reveals the measured spectral dependence of the switchable mirror’s reflectivity for right–handed circular polarized (RCP, +) and left-handed circular polarized (LCP, −) THz waves when VO_2_ is in its room temperature insulating phase (left) or its high–temperature conductive phase (right), where *R*_ij_ represents the i-polarized reflection from the j-polarized incidence. At room temperature, the proposed mirror behaves as an LCP mirror at its resonance 0.70 THz. It reflects the LCP incidence without changing its handedness (high *R*_--_ and low *R*_+-_), while absorbing the RCP incidence (low *R*_++_ and low *R*_-+_). Under thermal management, the insulator-to-metal transition of the VO_2_ film reverses the handedness of the conductive chiral split rings. Thus, the proposed mirror turns to an RCP mirror that reflects the incident RCP without handedness change (high *R*_++_ and low *R*_-+_) while absorbing LCP (low *R*_--_ and low *R*_+-_). Under thermal management, switching from a THz chiral mirror to a chiral mirror of opposite handedness, a conventional mirror, or a handedness-preserving mirror was demonstrated. Similar findings have also been reported with the structured VO_2_ incorporated into a metallic metasurface as an extension of the split ring resonators that dynamically controlled the near-field intensity [109] and switchable-focusing planar lens [75]. In contrast to the above lengthening strategy, the structured VO_2_ film could be used to shorten the metallic resonators of the THz metasurface based on the phase transition. An ultrathin quarter-wave plate, as shown in Figure 9b, is a hybrid complementary metasurface composed of asymmetric Cu cross-shaped-slot arrays, with the VO_2_ pads inserted at the end of the cross-shaped slots [110]. Increasing the operation temperature well above the phase transition temperature, the Cu cross-shaped slot was effectively shorted, developing a tunable quarter-wave plate from 0.468 to 0.502 THz. In addition to the lengthening and shortening strategies, connecting both ends of the metallic resonators by a VO_2_ inclusion was also an effective approach to achieve dynamically controlled THz metasurfaces. Figure 9c demonstrated a building block of the temperature-controlled three-dimensional-chiral metasurface, which was composed of pairs of identical and mutually twisted gold SRRs in parallel planes sandwiched in between the square VO_2_ rings (all the structures were supported by two pieces of sapphire at both ends) [53]. At room temperature, the three-dimensional chiral structure exhibited pronounced optical activity and a negative refractive index, since the VO_2_ film was in an insulating state. During the phase transition of the VO_2_ structure, the two ends of the gold SRRs were bridged gradually and finally turned out to be a fully connected square ring without any chirality, resulting in the absence of optical activity and a positive refractive index. It was the connection effect between the VO_2_ film and gold SRRs that switched the microstructure resonance from *LC* to the dipole resonance, initializing the dynamic modulation of the THz chiral metasurface. In addition, the hybrid metasurface composed of VO_2_-connected metallic SRRs were also produced via dynamically controlled asymmetric transmission [55] and holography [56]. In the above design strategy of tailoring the equivalent resonant microstructure of metasurfaces, the phase-transitioned metallic VO_2_ pattern turned out to be a part of the equivalent resonator through lengthening, shortening, or connecting the metallic structure of metasurfaces, thus leading to the thermally triggered switching operation.

Finally, we will discuss the fourth strategy of the thermally triggered THz metasurfaces: modifying the electromagnetic property of the VO_2_ patterns themselves. The temperature-induced insulator-to-metal phase transition of the VO_2_ film increased the conductivity up to 10^5^ S/m, which was comparable to that of metal (~10^7^ S/m). Thus, subwavelength microstructures made from metallic VO_2_ films support plasmon resonance as well, which can be used to construct a VO_2_ metasurface without any metallic component. The VO_2_ metasurfaces have received a lot of attention as they not only can be actively modulated, but also avoid a complex composite design [60,75,111]. As shown in Figure 9d, the VO_2_ cut wires were patterned on silica glass to construct a dynamic THz metasurface [60]. At room temperature (300 K), the measured results demonstrated that the VO_2_ metasurface exhibited a remarkable transparency exceeding 84% without any resonance performance, as shown in Figure 7e, since the insulating VO_2_ bars were almost transparent to the THz wave ranging from 0.2 to 1.65 THz. With the increasing operation temperature, the VO_2_ cut wires were gradually turned to be metallic bars supporting dipole resonances due to the phase transition. When the temperature was increased to 340 K, the VO_2_ metasurface exhibited a strong resonance around 0.6 THz with the transmission dropping to a minimum of 17%, resulting from the strong dipole resonance of the conductive VO_2_ cut wires. In this case, a dynamically controlled VO_2_ metasurface was developed through modifying the electromagnetic property of the VO_2_ microstructure under thermal management. In addition, the VO_2_ wire-grid metasurfaces were also a switchable THz polarizer with a polarization modulation depth reaching 63% [111]. However, there was still a certain conductivity difference between the conductive VO_2_ and metals; therefore, we must accept the tradeoff between the dynamic modulation and operational performance.

Thermal excitation is one of the most commonly used global excitation methods in VO_2_ hybrid THz metasurfaces. Although thermally triggered THz VO_2_-based metasurfaces undergo certain limitations in terms of the modulation rate and energy consumption, they can avoid the complex electrode design in the electrical excitation mode and the complex equipment requirements in the optical excitation process. Therefore, thermally triggered VO_2_ film-based THz metasurfaces are very promising from the device application perspective.

### 3.2. Electrically Triggered VO_2_ Film-Based THz Metasurfaces

**Figure 10:**
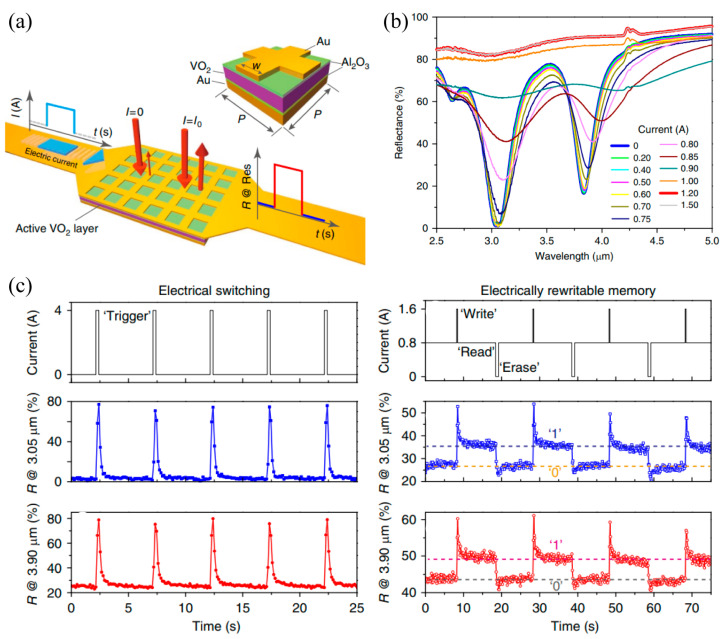
(**a**) Three-dimensional illustration of the metamaterial device consisting of a sandwich system with a 100 nm thick patterned-mesh top gold (Au) layer, a 260 nm thick active VO_2_ layer, and an optically thick (200 nm) Au ground plane. A 50 nm thick Al_2_O_3_ layer was applied in between both the gold and VO_2_ interfaces for optimized device performance. (**b**) Measured reflection spectra for various intensities of applied electrical current. A drastic but continuous spectrum tuning was achieved before saturation. (**c**) Observation of electrical switching of reflectance at the two resonance modes and the electrically rewritable memory effect in the meta-device [79]. “Reprinted/adapted with permission from Ref. [79]. 2016, Springer Nature”.

There have been significant studies on the electrically triggered VO_2_ film-based THz metasurfaces and how electrical excitation can also enable a phase transition in the VO_2_ film. In contrast to the global heating of thermal excitation, electrically controlled metasurfaces actuate the phase transition by inducing local heating precisely as the current flows through the VO_2_ film or the metal microstructures of the hybrid metasurfaces, which exhibits advantages in energy saving and device integration. Thus, electrically controlled hybrid VO_2_-based metasurfaces have attracted enormous attention in the THz regime. Here, we will introduce the recent progress in electrically triggered VO_2_-based THz metasurfaces categorized according to their design strategy. Firstly, we will present the VO_2_-based THz metasurface with respect to switching the operation mode by integrating a single continuous VO_2_ film. As shown in Figure 10a, the proposed metasurface absorber was composed of two continuous metallic layers sandwiching an Al_2_O_3_-VO_2_-Al_2_O_3_ spacer [79]. The top gold mesh layer was extended and connected to two external electrodes which not only supported optical resonance but also provided electrical heating via the Joule thermal effect. The reflection of the incident light at normal incidence would be immediately tuned as a function of the electrical current flowing through the layer triggering the phase transition in the active VO_2_ layer. At room temperature, the proposed metasurface behaved as a dual-band absorber around the wavelength of 3.90 and 3.05 μm, as shown in Figure 10b. An electric-switched operation mode from the absorber to the reflector has been observed by applying a rectangular-shaped current flow from 0 to 1.5 A, leading to the phase transition of the VO_2_ film by inducing local heating as it flowed through the top gold mesh layer. In this case, the metallization of the VO_2_ film completely destroyed the structure arrangement of the absorber on the met surface, causing the absorption effect at the two resonant frequencies to disappear completely as the transmission modulation exceeded 80% and 65% around 3.05 and 3.90 μm, respectively. Figure 10c demonstrated an electrical switching effect of the proposed hybrid metasurface. The time-resolved reflectance at the sub-second time scale was established when the metasurface was triggered by electrical current pulses with a width of 0.25 s. Notably, the memory metasurfaces have ignited interest in searching for unique performances, such as resonance tuning, information storage, and bistability. Such devices can be configured between the binary states of “0” and “1” with a long-lasting modification of electrically impulsive stimuli, as shown in Figure 8c. Resulting from the hysteresis performance of the VO_2_ film, photonic memory effect was initiated in this VO_2_-based hybrid metasurface with a bias current of 0.8 A applied to achieve the maximum hysteresis. This included an additional 0.25 s (0.75 s) current pulse of 0.8 A (−0.8 A) applied to “write” (“erase”) the rewritable photonic memory of the reflection signals. In the above design strategy of the switching operation mode, the VO_2_ film was isolated from the microstructure by the Al_2_O_3_ spacer, causing no influence to occur on the microstructure resonance directly. During the phase transition, the continuous VO_2_ film just behaved as a thin film with high transmission or high reflection.

**Figure 11:**
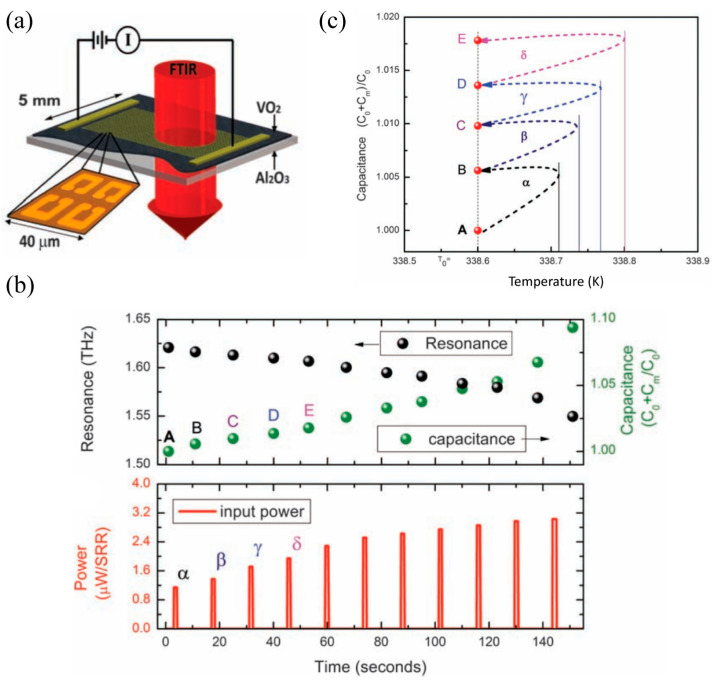
(**a**) Meta-device consisted of a gold SRRs array lithographically fabricated on a VO_2_ film. The electrodes were attached, allowing in-plane current-voltage relation transport, and the device was mounted to a temperature-control stage. (**b**) Successive modification of the resonance of the hybrid metamaterial was achieved using sequential transient 1-s electrical pulses of increasing power. These modifications persist until the device was thermally reset and have been measured to be stable over 20 min. (**c**) Operation of memory capacitance was explained via hysteretic phase transition [77]. “Reprinted/adapted with permission from Ref. [77]. 2009, American Association for the Advancement of Science”.

Subsequently, we will introduce the second strategy of electrically triggered THz metasurfaces: controlling the dielectric environment of the metallic microstructure by combining with the VO_2_ continuous film. In contrast to the first design strategy, directly patterning a metallic metasurface on the VO_2_ film will reduce the distance between them significantly. In this situation, the microstructure resonance will be significantly influenced by the phase transition of the VO_2_ film and the electromagnetic response of the metasurface could be electrically modulated. As shown in Figure 11a, a gold SRRs array was patterned on a VO_2_-Al_2_O_3_ substrate to implement a persistent frequency tuning of the THz memory metasurface [77]. Due to the large thermal mass of the continuous VO_2_ film and the limited local heating capacity of the current pulse, the sample was preset at 338.6 K via the hot plate due to the hysteresis effect at this temperature being most significant. In this case, the power flow promoted the phase transition effect accompanied by an increasing permittivity of the VO_2_ film and a rising capacitance in the SRRs, as shown in Figure 11b, which contributes to a successive red-shift resonance frequency and increasing on-resonance transmission. The first four excitation power α, β, γ, and δ is 1.1, 1.4, 1.7, and 2 μW/SRR, respectively. The frequency tuning and on-resonance transmission modulation depth was 0.4 THz and 6%, respectively, caused by the response of thermal excitation. Furthermore, a net change in the SRR capacitance was also achieved based on the hysteresis of the VO_2_ phase transition leading to a five-state photonic memory response with the electrical excitation response time less than 25 ms, as shown in Figure 11c, where A, B, C, D, and E represent the total capacitance of each SRR after electrical excitation. In the above design strategy of controlling the dielectric environment of the resonant microstructure, the VO_2_ continuous film behaved as an external disturbance. The Ohmic heat effect resulted from the electric current-induced phase transition of the VO_2_ film to tune the microstructure resonance.

**Figure 12:**
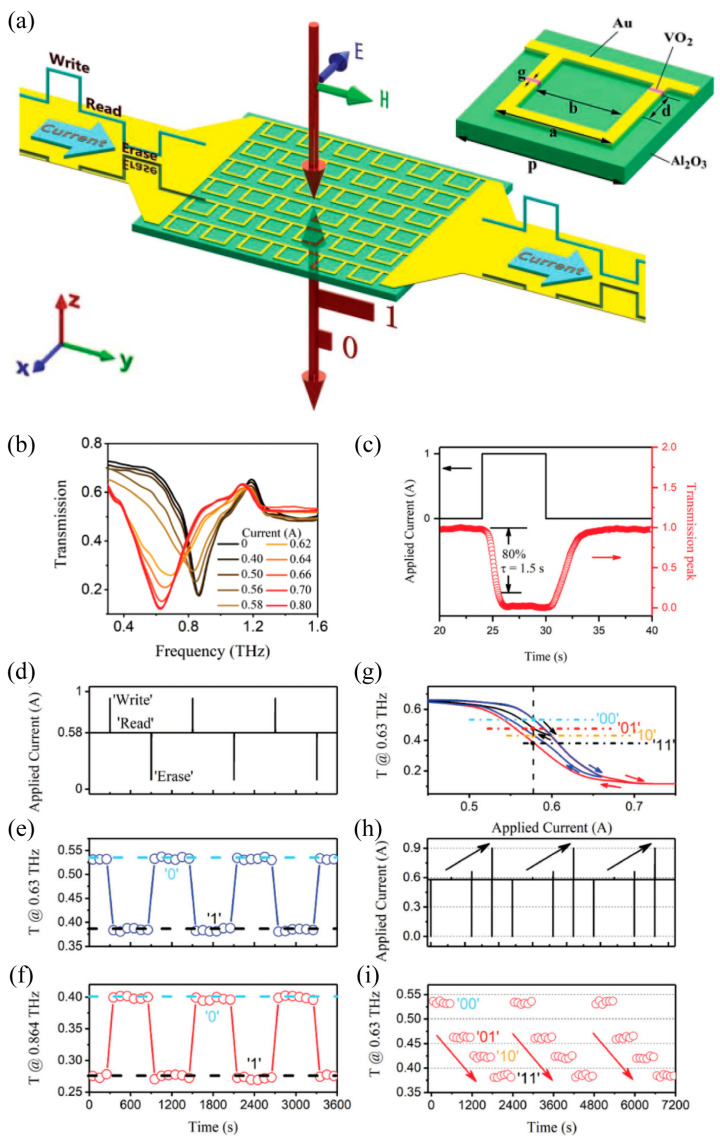
(**a**) Schematic view of the active hybrid metasurface. The normal incident THz wave propagated along the *z*-direction with its electric field polarized along the *x*-direction. The unit cell of the metasurface was depicted in the inset, where *p* = 100 µm, *a* = 70 µm, *b* = 54 µm, the size of the gaps was *g* = 3 µm, and the asymmetry between the two gaps was *d* = 20 µm. (**b**) Experimental transmission spectra as a function of the applied current. (**c**) Trigger signal (black, left axis) and monitored transmission peak (red, right axis) within one modulation cycle. (**d**) A bias current of 0.58 A was applied to achieve the maximum hysteresis. Additional write input (1 A, 1 s) and erase input (0 A, 2 s) were applied to switch between the “0” and “1” states of the transmission signal. (**e**,**f**) Timing diagrams of the transmission amplitudes measured at 0.63 and 0.864 THz, respectively. (**g**) Three hysteresis loops for the metasurface at 0.63 THz. The cyan, magenta, yellow, and black circles at 0.58 A represent the data states which are denoted as “00”, “01”, “10”, and “11”. (**h**) A bias current of 0.58 A was applied to achieve the maximum hysteresis. Additional current pulses were 0 (2 s)–0.58–0.6 (1 s)–0.58–0 (2 s)–0.66 (1 s)–0.58–0 (2 s)–0.9 (1 s)–0.58 A. (**i**) Timing diagram of the transmission amplitude at 0.63 THz corresponding to the three hystereses in (**g**) [59]. “Reprinted/adapted with permission from Ref. [59]. 2018, WILEY-VCH Verlag GmbH & Co. KGaA, Weinheim”.

On the contrary, the high reflection of the metallic continuous VO_2_ films limited the potential modulation depth in the transmission metasurface. In addition, the structured VO_2_ patterns exhibited a much smaller thermal mass and a much faster switching rate since the local thermal energy was unnecessarily dissipated to cover the entire metasurface. Thus, the structured VO_2_ patterns were much more suitable than continuous films for hybrid metadevices. Here, we will introduce the recent progress in the third strategy of electrically triggered THz metasurfaces: tailoring the equivalent resonant microstructure by connecting metallic microstructures with the structured VO_2_ patterns [12,13,59,85,112,113,114]. As shown in Figure 12a, the gold asymmetric split ring resonators (ASRRs) with the VO_2_ patterns integrated within the two side gaps were deposited on a sapphire substrate, and the continuous ASRRs array was adhered to an external circuit to construct an electrically controlled multifunctional tunable metasurface based on the phase transition of the VO_2_ pattern [59]. As shown in Figure 12b, increasing the current injection led to a phase transition of the VO_2_ pattern from the insulator to the metal and the resonance frequency shifted from 0.91 to 0.64 THz (i.e., frequency tuning of 0.27 THz) with the threshold current at about 0.4 A (0.8 V) and the saturated current at 0.75 A (1.5 V). In particular, a large modulation depth of 54% was achieved at 0.63 THz, which revealed promising applications in the photonic memory function. A bias current of 0.58 A was employed to maximize the hysteresis and it was set as the “read” input. The excitation of the “write” input (1 A, 1 s) and the “erase” input (0 A, 2 s) were demonstrated in Figure 12d. In relation to the excitation, the timing diagrams of transmission amplitudes measured at 0.63 THz and 0.864 THz are provided in Figure 12e and Figure 12f, respectively, where the circles represent the amplitude transmission. The timing diagrams of the on-resonance transmission between Figure 12e,f exhibited a contrasting flip-flop switching at 0.63 THz and 0.864 THz, respectively. Furthermore, four different transmissions at 0.58 A were obtained based on the hysteretic property of VO_2_, as shown in Figure 12g, then a four-state memory transmission, as shown in Figure 12i, was developed under the excitation of the current sequence, as shown in Figure 12h. Benefiting from the low thermal mass of the VO_2_ patterns and the high-quality factor resonance of ASRRs, the proposed multifunctional metasurface exhibited the following advantages: operating at room temperature without the need of additional bias temperature achieving a much greater modulation depth, which enabled a more remarkable binary memory response and significantly reduced the voltage, current, and power (225 μW for each VO_2_-ASRR unit cell) required for external electrical excitation. Though the energy consumption of 225 μW for each VO_2_-ASRR unit cell was larger than that of 1.2~3.0 μW/SRR in Ref. [97], this hybrid metasurface can be electrically switched solely at room temperature, eliminating the requirement for an additional bias temperature. Furthermore, the rise and fall time of the electrically controlled time-domain transmission peak were 2.2 s and 4 s, respectively, as shown in Figure 12c, which can be further reduced by increasing the external excitation current. This work demonstrated a novel approach to constructing the VO_2_-based hybrid metasurface with the merit of highly integrating, energy saving, large modulation depth, and fast switching. Based on the same design idea, a series of THz metal-VO_2_ metasurfaces were predicted to initiate electrically triggered mode switching (from one bonding dimer plasmonic mode to a charge transfer plasmonic mode and a screened bonding dimer plasmonic mode simultaneously) [85], the broadband quarter-wave plate [113], and the plasmonic-induced transparency resonance [114]. Recently, this type of electrically triggered metal-VO_2_ THz metasurface was developing towards large-scale, multi-level, multi-functional, and intelligent application including the programmable spatial wave modulators [12] and the self-adaptive intelligent metasurfaces [13]. In the above design strategy of tailoring the equivalent resonant microstructure of metasurfaces, the phase-transitioned metallic VO_2_ film behaved as part of the equivalent resonant microstructure in order to connect the metal microstructure of metasurfaces, resulting in the initialization of the electrically triggered switching operation.

Noticeably, there was less discussion on the fourth strategy of the electrically triggered THz metasurface involving the modification of the electromagnetic property of the VO_2_ metasurface microstructures. There are three possible reasons contributing to this situation. First of all, the ability of the electrically triggered local heating method to achieve phase transition is not strong enough, especially compared with the global heating method. Second, in contrast to the small-sized VO_2_ patterns which are inserted into the metal metasurface microstructures, the VO_2_ unit cell array exhibits higher thermal mass; thus, the electrically triggered phase transition of the VO_2_ metasurface is more challenging. Third, the metal circuit wiring for electrodes exerts a strong influence on the VO_2_ metasurface because there are two orders of magnitude differences in electrical conductivity between the metallic VO_2_ and the metallic wiring materials of electrodes.

Electric excitation is a convenient and low-cost approach to switch the response of the VO_2_-based THz metasurface by inducing local heating as the current flows through the VO_2_ film or metal microstructures. Additionally, the electrically triggered THz metasurfaces based on the VO_2_ film exhibit the merit of highly integrating, energy saving, large modulation depth, and fast switching. However, the repetition rates will always be limited to the kilohertz level.

### 3.3. Optically Triggered VO_2_ Film-Based THz Metasurfaces

**Figure 13:**
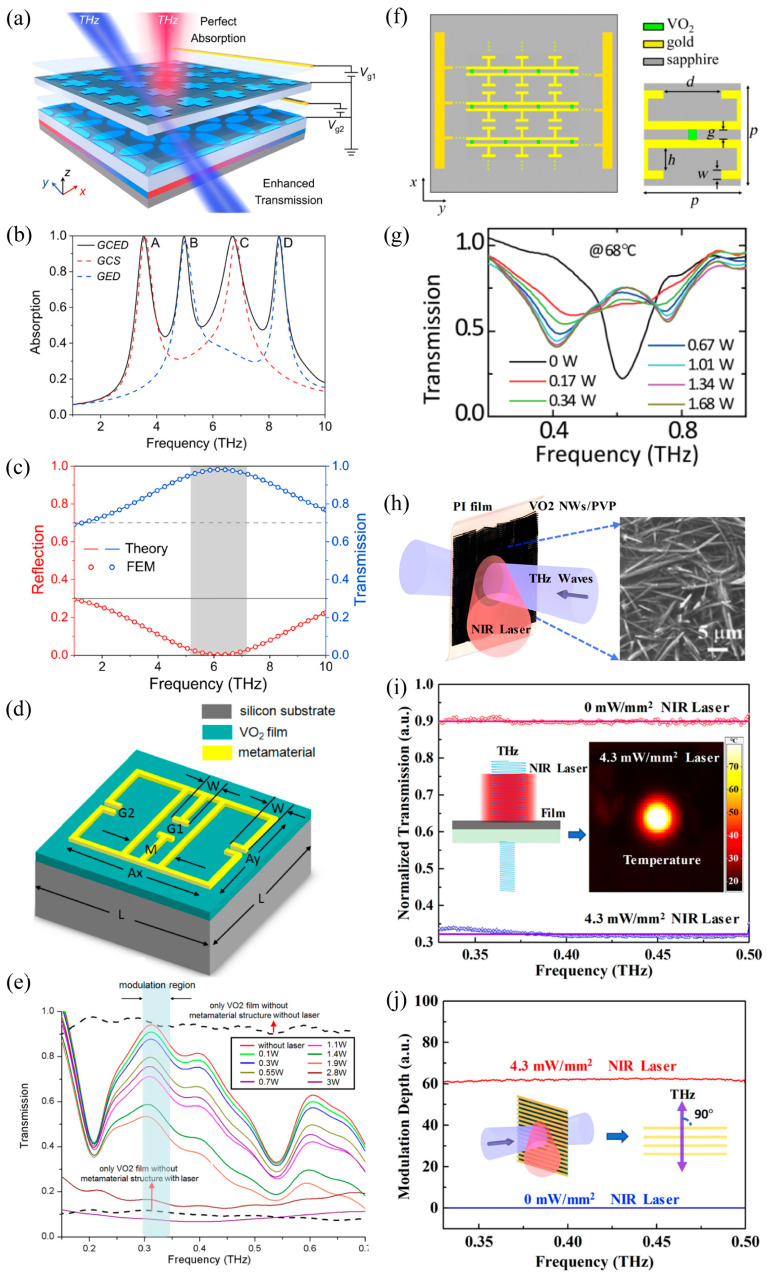
(**a**) Schematic diagram of the multifunctional metasurfaces with switchable functionality from perfect absorption to enhanced transmission. (**b**) Absorption spectra of the GCED (black solid line), GCS (red dashed line), and GED (blue dashed line) structures. (**c**) Reflection and transmission spectra were obtained from the FEM simulations (hollow circles) and theoretical calculations (solid curves). The solid and dashed gray lines denote the reflection and transmission, respectively, of the bare Si substrate. The gray shaded region depicts the frequency band for the *R* < 3% criterion [41]. “Reprinted/adapted with permission from Ref. [41]. 2022, American Chemical Society”. (**d**) Structure of DMVS with the 3D model of one unit. (**e**) Fourier transform of time-domain waveforms was obtained based on the characterization by THz-TDS [115]. “Reprinted/adapted with permission from Ref. [115]. 2014, The Optical Society of America”. (**f**) (**Left**): Schematic diagram of metasurface embedded with 200 nm thick VO_2_ film (green color). (**Right**): Geometric parameters of unit cell of the MM, *p* = 100 µm, *g* = 10 µm, *w* = 8 µm, *d* = 60 µm, and *h* = 22 µm. (**g**) THz transmission of the VO_2_-embedded hybrid MM under an optical stimulus at 68 °C [85]. “Reprinted/adapted with permission from Ref. [85]. 2019, American Physical Society”. (**h**) Prepared VO_2_/PVP film and the schematic of optical-pump THz-probe spectroscopy characterization. (**i**) Normalized THz transmission of the VO_2_/PVP film under NIR laser irradiation of different laser power densities (0 and 4.3 mW/mm^2^), and solid lines are fitted to the experimental data. The size of the circular spot in the temperature map was slightly larger than that of the NIR laser beam spot (spot diameter ∼6 mm). The insets are the experimental setup and temperature profile of the composite film in the NIR laser beam spot area under laser irradiation of 4.3 mW/mm^2^. (**j**) Maximum modulation depth of THz waves via the tunable flexible THz polarizer at the polarization angle of 90° under NIR laser irradiation of 4.3 mW/mm^2^ [116]. “Reprinted/adapted with permission from Ref. [116]. 2021, American Chemical Society”.

The phase transition triggered both thermally and electrically on the VO_2_ film were mainly resulted from the thermal effect. Therefore, the repetition rate was estimated to be in the order of kilohertz, which still needs to improve for the increasing information and communication demands. Fortunately, the optically triggered VO_2_ film with photon energy larger than the insulation phase gap by about 0.7 eV exhibits a subpicosecond response, which can largely alleviate the above dilemma. Thus, great effort has been made and valuable progress has been achieved in optically triggering the THz metasurface based on the VO_2_ film. The first strategy for optically triggered THz metasurfaces involves switching the operation mode by integrating a single continuous VO_2_ film. As shown in Figure 13a, the proposed hybrid metasurface was composed of two graphene microstructure arrays separated by a SiO_2_ spacer and a VO_2_ film via the support of a Si substrate [41]. When there was no light trigger, the VO_2_ film was in an insulating state and the proposed metasurface exhibited a unit transmission with the graphene Fermi energy level fixed at 0. When the metasurface was triggered by a continuous 532 nm laser, the VO_2_ film was changed into a metal mirror. In this case, this hybrid metasurface behaved as a quad-band absorber, as shown in Figure 13b, with the Fermi energy levels set as 0.43 and 0.57 eV for the top and bottom graphene layer, respectively. Absorption spectra of the entire metasurface structure (GCED) exhibit four absorption peaks (A, B, C, and D) with absorption efficiencies of 99.85, 99.74, 99.79, and 99.56% at frequencies of 3.53, 4.98, 6.70, and 8.36 THz, respectively. More specifically, the absorption peaks A and C are derived from the resonance absorption of graphene cross-shaped resonator array (GCS), and the absorption peaks B and D are derived from the resonance absorption of graphene elliptical nanodisk array (GED). Therefore, switching between perfect absorption and complete transmission was demonstrated by optically stimulating the VO_2_-based metasurface, as shown in Figure 13c. In the above design strategy of the switching operation mode, the VO_2_ film was far away from the microstructure and exerted little influence on the resonance of the microstructure. During the phase transition, the continuous VO_2_ film just behaved as a thin film with high transmission or high reflection.

Furthermore, we will review the second strategy of optically triggered VO_2_-based THz metasurfaces regarding the control of the dielectric environment of the metallic microstructure by combining continuous VO_2_ film [115,117,118,119]. In contrast to the first design approach, directly patterning the metallic metasurface on the VO_2_ film will eliminate the gap between the two layers. In this case, the resonance of the microstructures will be intensively influenced by the phase transition of the VO_2_ film and the electromagnetic response of the metasurface can be optically modulated. As shown in Figure 13d, the proposed dual-resonance metasurface unit cell was composed of a silicon substrate, continuous VO_2_ film, and an Al microstructure layer from bottom to top [115]. The sample was characterized by THz-TDS under the excitation of a light beam from an oblique angle. The simulated and measured results demonstrated that the proposed metasurface exhibited a transmission band from 0.28 to 0.37 THz as shown in Figure 13e, which is around the first atmosphere window of 0.34 THz. By obliquely illuminating the hybrid metasurface via a continuous 808 nm laser, the phase transition of the VO_2_ film modified the resonant environment of the aluminum microstructure, directly resulting in a continuous modulation on the transmission band of the metasurface. The measured results showed that the modulation depth exceeded 80% and the modulation rate reaches values up to 1 MHz. This work demonstrated a simple and universal approach for the design of ultrafast and large-depth THz modulators. Based on a similar design approach, ultrafast all-optical modulation or the switching of THz transmission have been developed under the excitation of a continuous 532 nm laser [117] and a pulsed 780–730 nm laser [118]. In the above studies, the strongly localized electric field of nanoslot resonators led to an enhanced nonlinear response of the VO_2_ film, which resulted in a significant reduction in the pumping power or energy. Furthermore, optically triggered phase transition of the VO_2_ film was also employed to modify the resonance environment of the dielectric metasurface to initialize the state conversion in the THz regime [119]. In the above design strategy of controlling the dielectric environment of metallic resonant microstructures, the VO_2_ continuous film located in the resonance region of the metallic metassurface behaved as an external disturbance. The optically induced phase transition of the VO_2_ film was applied to suppress the resonance of the metallic microstructure.

Additionally, there have been extensive studies on the optically triggered hybrid THz metasurface composed of metallic microstructures and the VO_2_ patterns. Next, we will discuss the third strategy of the optically triggered THz metasurface which involves tailoring the equivalent resonant microstructures by connecting metallic microstructures with the structured VO_2_ patterns [59,85]. By integrating metallic resonators with the VO_2_ structures, the hybrid metasurface exhibited a tunable resonance which can be seen via the equivalent resonant geometry that can be dynamically reconfigured due to the phase transition of VO_2_. As shown in Figure 13f, the proposed metasurface unit cell was composed of two folded gold wire structures connected by an integrated VO_2_ pattern forming the optically triggered THz dimer array hybrid metasurface [85]. When the hybrid metasurface operated at room temperature and without optical excitation, the transmission spectra exhibited a remarkable bonding dimer plasmonic mode, as shown in Figure 13g. As the signal of the THz TDS system was very weak at the low frequency limit of 0.2 THz, the background noise exerted a strong influence on the measured result, resulting in a transmission greater than 1 around 0.2 THz. By increasing the operation temperature to 68 °C, a phase transition consequently occurred in the VO_2_ patterns under the excitation of the 800 nm and a 1 kHz pulsed laser. This rapid phase transition was completed in only 4 ps. Further increase in the pumping power to 1.68 W resulted in the VO_2_ patterns turning into the metallic state. As a result, the transmission spectra split from a single bonding dimer plasmonic mode to a charge transfer plasmonic mode and a screened bonding dimer plasmonic mode simultaneously, as shown in Figure 13g. It was noted that in this optically triggered THz metasurface, the sample operation needed to set a bias temperature (such as 60 °C, 64 °C, and 68 °C) to reduce the threshold of the energy density of the optical excitation pulses. This was due to the fact that the optical pump power threshold was reducing with the increasing bias temperature and it was close to 0 W at the critical temperature of 68 °C. Based on the same design approach, the optically triggered ultrafast amplitude [59] and phase [101] modulation of THz metasurfaces were developed under the excitation of a 800 nm femtosecond laser. In the above design strategy of tailoring the equivalent resonant microstructure of metasurfaces, the phase-transitioned metallic VO_2_ pattern turned out to be a part of the equivalent resonator via its integration to the metallic structure of the metasurface, initializing an optically triggered switching operation.

Lastly, we will analyze the fourth strategy of the optically triggered THz metasurface: the modification of the electromagnetic properties of the patterned VO_2_ films. This was represented by the all-optically triggered THz flexible device, as shown in Figure 13h [116]. The VO_2_ nanowires/poly-(vinylpyrrolidone) (PVP) were prepared on polyimide (PI) by spin coating. The structured VO_2_ nanowires exhibited a random and uniform arrangement. The fabricated VO_2_/PVP sample appeared black since it has a strong absorption over the broadband spectrum (400–900 nm) of light. Thus, illuminating the VO_2_/PVP film with a continuous 808 nm laser will enable a phase transition of the VO_2_ nanowires based on the photothermal effect. In the absence of optical excitation, the VO_2_/PVP film revealed a high transmission up to 90% from 0.33 to 0.5 THz, as shown in Figure 13i. By increasing the laser power density to 4.3 mW/mm^2^, the transmission dropped dramatically to approximately 30%, due to the phase transition of the VO_2_ nanowires. Although the VO_2_ nanowires were randomly arranged and did not form a metasurface, they could still manipulate the amplitude of the THz waves. Furthermore, an optically tunable and highly flexible THz polarizer based on the VO_2_/PVP composite film was demonstrated. As shown in Figure 13j, the proposed polarizer was composed of a gold wire grid pattern, a PI film spacer, and a VO_2_/PVP film. In the absence of optical excitation, the proposed sample behaved as a THz metal polarizer. By illuminating the sample from the front side, the exposed VO_2_ was transformed into a metallic state and the VO_2_/PVP film was then reconfigured into an equivalent grating structure. Integration of the gold wire grid pattern and metallic VO_2_ wire grating has led to a large modulation depth exceeding 60%. In this case, the exposed VO_2_ strips formed a VO_2_ metasurface layer; therefore, photoexcitation of the VO_2_ grating was equivalent to modifying the electromagnetic properties of the VO_2_ metasurface unit cell. This work demonstrated a new method to construct metallic VO_2_ metasurfaces using metal microstructure patterns to block the excitation light and produce specific optical patterns.

Optical illumination is also an indispensable excitation method in the VO_2_-based THz metasurfaces. Although the optically triggered VO_2_-based THz metasurfaces experienced certain shortcomings in terms of the high excitation threshold, low modulation depth, and expensive laser equipment, they are promising for application that require ultrafast modulation and time-resolved measurement.

### 3.4. THz Field Triggered VO_2_-Based THz Metasurfaces

**Figure 14:**
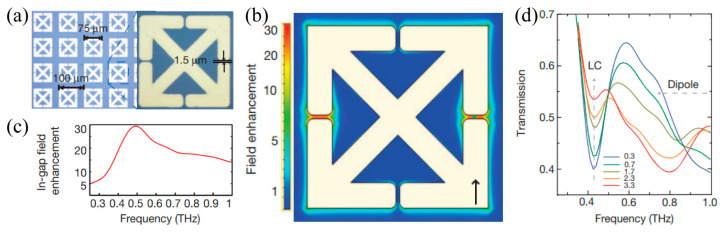
(**a**) Optical image of metamaterial SRRs deposited on VO_2_/sapphire with a 1.5 μm gap in the SRR unit cell. (**b**) Resonant field enhancement as a function of position. (**c**) Frequency-dependent in-gap field enhancement. (**d**) Experimental data showing field-dependent nonlinear transmission spectra of SRRs on VO_2_ at 324 K for in-gap fields ranging from 0.3 to 3.3 MV cm^−1^ [122]. “Reprinted/adapted with permission from Ref. [122]. 2012, Macmillan Publishers Limited.”.

Recently, it has been demonstrated that THz nanogap structures in the metallic metasurfaces exhibit strong in-gap field enhancement exceeding three orders of magnitude [120,121] and these intensive THz field could reduce the Coulomb-induced potential barrier for carrier transport, leading to a phase transition of the in-gap VO_2_ film. In addition, the nanogap structures were ultra-sensitive to the gap environment. The nonlinear spectral response resulting from the phase transition of the VO_2_ film could be effectively characterized. As shown in Figure 14a, the gold SRRs with a small gap size of 1.5 μm were deposited on the VO_2_ film to form a THz metasurface on a sapphire substrate [122]. The simulated near-field distribution at the *LC* resonance frequency demonstrated an electric field enhancement in the two horizontal SRR gaps by more than 27 times, as shown in Figure 14b,c. This field enhancement promoted a phase transition for the in-gap VO_2_ film, which was recorded and verified by the transmission spectra. As shown in Figure 10d, by increasing the in-gap field from 0.3 to 3.3 MV/cm, the on-resonance (at 0.41 THz) transmission increased significantly. The measured results indicated that with the increase in the in-gap field, the phase of the VO_2_ film was gradually changed to the metal state. As a result, the gaps in the SRRs were shorted gradually and the LC resonances were gradually suppressed. This was a unique approach that triggered the phase transition of the VO_2_ film by use of field enhancement occurring in the metasurface unit cell. Furthermore, the THz nanogap of the metasurface with a strong electric field enhancement has developed into a powerful platform for THz science and application research. Extensive studies have been conducted to reduce the phase transition temperature and hysteretic width of VO_2_ films based on the nanogap metasurface.

## 4. Conclusions and Outlook

The strongly correlated electric material VO_2_ experiences an insulator–metal phase transition when triggered by external physical excitations, such as heat, electric bias, and light waves. The ultrafast and vast conductivity modification occurring near room temperature endows the VO_2_ films to be advantageous in developing high-speed THz devices with a desired large modulation depth. Benefitting from the hysteretic properties of the phase transition loop, the VO_2_ films guarantee the ability to design THz photonic memory and storage devices. Many substantial studies have focused on the external stimuli triggered VO_2_-based hybrid THz metasurfaces. We systematically reviewed the latest progress on the external-field triggered THz metasurfaces that exploit the VO_2_ phase transition and classified those in the categories of stimuli. For each type of stimulant, four types of metasurface composite methods were summarized including switching the operation mode of metasurfaces, controlling the dielectric environment of metallic resonant microstructures, tailoring the equivalent resonant microstructures, and modifying the electromagnetic property of the VO_2_ metasurfaces’ unit cells. The design and reconfiguration of the VO_2_-based metasurfaces were discussed with a particular focus on the critical role of phase transition in the VO_2_ films during the dynamic modulation processes.

While significant progress on the VO_2_-based THz metasurfaces has been witnessed, there are still issues that need to be resolved. Firstly, the phase transition threshold needs to be further reduced. In the thermally triggered method, the phase transition temperature was 68 °C, which is more than 40 °C above room temperature, limiting its use in certain practical applications. Additionally, the electrical and optical triggering always requires a biased temperature or current setting to achieve a phase transition of VO_2_, which makes the triggering operation more complicated and cumbersome. Furthermore, a high-contrast modulation depth and broad hysteresis are expected. THz switches, memory, and storage devices put forward higher requirements for the modulation depth and hysteresis width of the VO_2_ film in order to establish a stable multistate in a large interval. As a final point, the comprehensive triggering performance for each type of excitation needs to be improved. It was noted that each type of excitation exhibited at least one serious drawback, such as the low modulation rate in thermal excitation, complex metal wiring in electrical excitation, and complicated and expensive laser equipment in optical excitation.

In order to further enhance the comprehensive performance of VO_2_-based meta-geometries and meta-devices, multilayered metasurfaces with enhanced control of longitudinal coupling of microstructures may break the constraints of most metasurfaces that solely utilize in-plane microstructure coupling. Also, VO_2_-based metasurfaces simultaneously triggered by multiple physical fields are promising in exploring novel coupling phenomena and physics. In conclusion, the integration of programming and intelligent algorithms into VO_2_-based meta-geometries is desired in developing active THz photonic devices, such as spatial light modulators and intelligent components. metasurfaces.

## Data Availability

Not applicable.
